# Inhibition of a type III secretion system by the deletion of a short loop in one of its membrane proteins

**DOI:** 10.1107/S0907444913002102

**Published:** 2013-04-11

**Authors:** Vladimir A. Meshcheryakov, Akio Kitao, Hideyuki Matsunami, Fadel A. Samatey

**Affiliations:** a Trans-Membrane Trafficking Unit, Okinawa Instiute of Science and Technology, Okinawa 904-0495, Japan; b Institute of Molecular and Cellular Biosciences, University of Tokyo, Tokyo 113-0032, Japan; c Japan Science and Technology Agency, Core Research for Evolutionary Science and Technology, Tokyo 113-0032, Japan

**Keywords:** FlhB, bacterial flagellum, type III secretion system, *Salmonella*, *Aquifex*

## Abstract

The membrane protein FlhB is a highly conserved component of the flagellar secretion system. It is composed of an N-­terminal transmembrane domain and a C-terminal cytoplasmic domain (FlhB_C_). Here, the crystal structures of FlhB_C_ from *Salmonella typhimurium* and *Aquifex aeolicus* are described at 2.45 and 2.55 Å resolution, respectively. These flagellar FlhB_C_ structures are similar to those of paralogues from the needle type III secretion system, with the major difference being in a linker that connects the transmembrane and cytoplasmic domains of FlhB. It was found that deletion of a short flexible loop in a globular part of *Salmonella* FlhB_C_ leads to complete inhibition of secretion by the flagellar secretion system. Molecular-dynamics calculations demonstrate that the linker region is the most flexible part of FlhB_C_ and that the deletion of the loop reduces this flexibility. These results are in good agreement with previous studies showing the importance of the linker in the function of FlhB and provide new insight into the relationship between the different parts of the FlhB_C_ molecule.

## Abstract 2

Crystal structures of the cytoplasmic domain of FlhB from *S. typhimurium* and *A. aeolicus* were solved at 2.45 and 2.55 Å resolution, respectively. The deletion of a short loop in the cytoplasmic domain of *Salmonella* FlhB completely abolishes secretion by the type III secretion system. A molecular-dynamics simulation shows that the deletion of the loop affects the flexibility of a linker between the transmembrane and cytoplasmic domains of FlhB.

## 1. Introduction   

The bacterial flagellum is a large complex structure that is used as a motility organelle by many bacteria. It consists of three main substructures: the basal body, the hook and the filament. Most of the flagellar proteins are localized outside the cell and are translocated across the cell membrane by the flagellum-specific secretion apparatus, which is evolutionarily and structurally related to the virulence type III secretion system (Aizawa, 2001 ▶; Blocker *et al.*, 2003 ▶). Protein export by the flagellar type III secretion system is highly regulated. The secretion system first exports rod/hook-type proteins until the hook reaches an appropriate length. The secretion system then switches substrate specificity from hook-type export to filament-type export (Minamino & Macnab, 1999 ▶; Hirano *et al.*, 2003 ▶). In *Salmonella*, the substrate-specificity switch is controlled by two proteins: FliK and FlhB (Kutsukake *et al.*, 1994 ▶; Hirano *et al.*, 1994 ▶; Williams *et al.*, 1996 ▶).

FlhB is an essential membrane protein of the flagellar type III secretion system. It consists of two domains: a hydrophobic N-terminal part (FlhB_TM_) that is predicted to contain four transmembrane helices and a C-terminal cytoplasmic domain (FlhB_C_) (Minamino *et al.*, 1994 ▶). The two domains are connected by a flexible linker. This linker is a highly conserved part of the FlhB protein and is essential for the type III secretion system (TTSS). Deletions or point mutations in the linker region completely abolish or significantly reduce secretion (Fraser *et al.*, 2003 ▶; Zarivach *et al.*, 2008 ▶). The wild-­type cytoplasmic domain of *Salmonella* FlhB undergoes autocatalytic cleavage between amino-acid residues Asn269 and Pro270 within a highly conserved NPTH sequence (Minamino & Macnab, 2000*a* ▶). This autocleavage is essential for the switching process (Fraser *et al.*, 2003 ▶; Ferris *et al.*, 2005 ▶). Mutation of Asn269 to Ala prevents cleavage and locks the export apparatus in the hook-type specificity state.

FlhB_C_ has been shown to interact with several soluble components of the TTSS: FliH, FliI, FliJ (Minamino & Macnab, 2000*b* ▶), the cytoplasmic part of the membrane protein FlhA (Zhu *et al.*, 2002 ▶) and the hook-length control protein FliK (Minamino *et al.*, 2004 ▶; Morris *et al.*, 2010 ▶). Interaction of FlhB with FliK has been suggested to be important for the substrate-specificity switching process (Ferris & Minamino, 2006 ▶). Cells with a deleted *fliK* gene produce an abnormally long hook, termed a ‘polyhook’, without any filament attached (Hirano *et al.*, 1994 ▶).

Several structures of the cytoplasmic domain of FlhB paralogues from the needle TTSS have been published (Zarivach *et al.*, 2008 ▶; Deane *et al.*, 2008 ▶; Wiesand *et al.*, 2009 ▶; Lountos *et al.*, 2009 ▶). However, no structural information is available for FlhB from the flagellar secretion system. Here, we describe crystal structures of the cytoplasmic domain of flagellar FlhB from two organisms: *Salmonella typhimurium* and *Aquifex aeolicus*.

## 2. Materials and methods   

### 2.1. Structure determination   

Details of the purification, crystallization and data collection of *Salmonella* and *Aquifex* FlhB_c_ have been described previously (Meshcheryakov *et al.*, 2011 ▶; Meshcheryakov & Samatey, 2011 ▶). Both structures were solved by multiwavelength anomalous diffraction (MAD) using the program *SHELXD* (Sheldrick, 2008 ▶). Initial protein models were built automatically with *Buccaneer* (Cowtan, 2006 ▶) from the *CCP*4 package (Winn *et al.*, 2011 ▶). The models were refined through an iterative combination of refinement with *REFMAC*5 (Murshudov *et al.*, 2011 ▶) and manual model building in *Coot* (Emsley *et al.*, 2010 ▶). In the case of *Salmonella* FlhB_C_, TLS refinement was performed in the final stages with two TLS groups per FlhB_C_ molecule (residues 229–269 and 270–353; Painter & Merritt, 2006 ▶). Structural figures were produced using *PyMOL* (http://www.pymol.org).

### 2.2. DNA manipulation and motility assay   

Mutations of *S. typhimurium flhB* carried by the plasmid pMM26 (Minamino & Macnab, 2000*a* ▶) were performed as described previously (Wang & Malcolm, 1999 ▶). For the motil­ity assay, freshly transformed *Salmonella* cells were directly inoculated as colonies into soft tryptone agar containing 0.35%(*w*/*v*) agar and incubated at 303 K.

### 2.3. Preparation of the whole-cell and culture-supernatant fractions and immunoblotting   

*Salmonella* MKM50 (Δ*flhB* strain) cells (Fraser *et al.*, 2003 ▶) carrying an appropriate plasmid were incubated at 310 K in LB medium containing 100 µg ml^−1^ ampicillin until the optical density OD_600_ reached 1.4–1.5. Aliquots of culture containing a constant amount of cells were centrifuged. Cell pellets were suspended in an equal volume of SDS loading buffer. Proteins in the culture supernatant were precipitated using 10% trichloroacetic acid and were suspended in SDS loading buffer. After SDS–PAGE, proteins were detected with anti-FlgE and anti-FliC antibodies using a WesternBreeze chromo­genic immunodetection kit (Invitrogen).

### 2.4. Molecular-dynamics simulations   

Molecular-dynamics (MD) simulations were performed using the *SCUBA* (*Simulation Codes for hUge Biomolecular Assembly*) program package (Ishida *et al.*, 2006 ▶). The AMBER ff99SB force field (Hornak *et al.*, 2006 ▶) was used for the protein. The simulated systems were solvated with SPC/E water molecules (Berendsen *et al.*, 1987 ▶) with 100 m*M* KCl in the periodic boundary separated by at least 12 Å from the FlhB_C_ molecule in the initial stage. After energy minimization and 0.27 ns MD simulation to adjust the temperature and pressure of the system to 300 K and 101 kPa with positional restraints, 40 ns MD simulation was performed without restraints in the canonical ensemble. The last 20 ns trajectory was used for the analysis. A shifted-force cutoff of real-space nonbonded energy was made at 12 Å and the particle-particle-particle-mesh (PPPM) method (Deserno & Holm, 1998 ▶) was employed for electrostatic energy calculation in Fourier space. Integration of the equation of motion was carried out using the multi-time-step method *XO-RESPA* (Martyna *et al.*, 1996 ▶) in the canonical ensemble. Integrations of fast (bond and angle), medium (torsion and real-space nonbonded) and slow (Fourier-space nonbonded) energy terms were performed every 0.5, 1.0 and 2.0 fs, respectively.

### 2.5. Accession numbers   

Atomic coordinates and structure factors have been deposited in the PDB with accession codes 3b0z and 3b1s for *Salmonella* and *Aquifex* FlhB_C_, respectively. The structures reported here are explained in interactive three dimensions at http://proteopedia.org/w/Samatey.

## 3. Results and discussion   

### 3.1. Flagellar FlhB_C_ structure description   

The *Salmonella* FlhB_C_ (SalFlhB_C_) and *Aquifex* FlhB_C_ (AquFlhB_C_) structures were solved by multiwavelength anomalous diffraction (MAD) using selenomethionine derivatives (Meshcheryakov *et al.*, 2011 ▶; Meshcheryakov & Samatey, 2011 ▶; Table 1 ▶).

The SalFlhB_C_ and AquFlhB_C_ crystals belonged to different space groups: *P*4_2_2_1_2 and *C*2, respectively. In the case of the AquFlhB_C_ crystal there are three protein molecules in the asymmetric unit. The three molecules in the asymmetric unit are very similar, with r.m.s.d.s on pairwise superposition in the range 0.40–0.76 Å. Each molecule consists of two polypeptide chains resulting from proteolytic cleavage after Asn263. In all molecules no electron density was observed for residues 213–231 at the N-terminus; depending on the molecule, two to six residues at the C-terminus were disordered.

**Figure 1:**
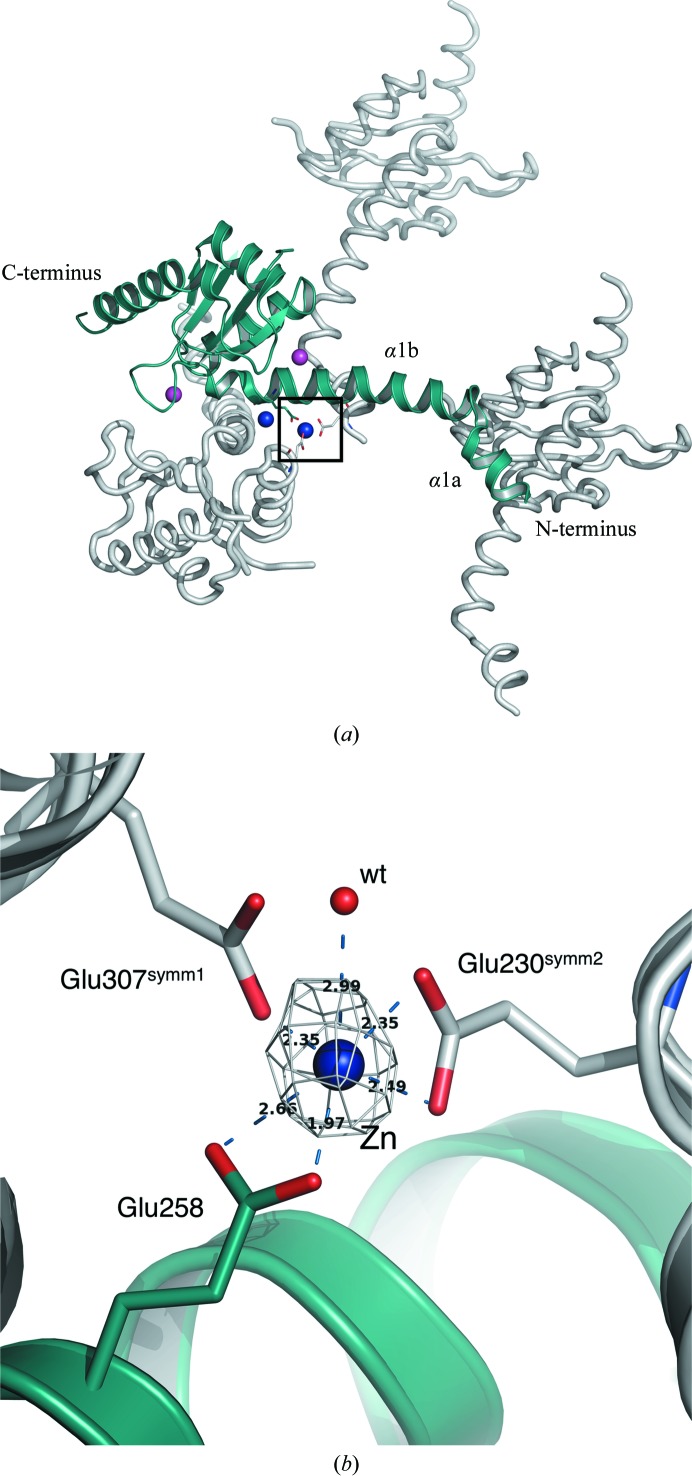
Molecular packing in the crystal of *Salmonella* FlhB_C_. (*a*) The asymmetric molecule and symmetry-related molecules are displayed in green and grey, respectively. Sodium ions are represented as magenta spheres and zinc ions are shown as blue spheres. (*b*) Rotated enlarged view of the zinc-binding site (black box in Fig. 1 ▶ *a*). The *F*_o_ −*F*_c_ electron-density map is displayed in grey at a contour level of 5σ and was calculated without a Zn atom.

In the case of SalFlhB_C_ the final model comprises residues 229–353 out of 219–383 in the crystallized protein, with a cleavage after Asn269. No electron density was observed for residues 219–228 and 354–383. The model of *Salmonella* FlhB_C_ includes two Zn^2+^ ions and two Na^+^ ions (Fig. 1 ▶ *a*). All of these atoms mediate intermolecular interactions in the crystal lattice. Zn^2+^ was added to the crystallization solution and was necessary to obtain well diffracting crystals. Analysis of the crystallographic packing shows that one of the zinc ions coordinates three glutamate residues from three symmetry-related SalFhB_C_ molecules: Glu230, Glu258 and Glu307 (Fig. 1 ▶ *b*). This interaction fixes N-terminal helix α1, which is one of the most flexible parts of *Salmonella* FlhB_C_ (see below), between two symmetrical molecules.

**Figure 2:**
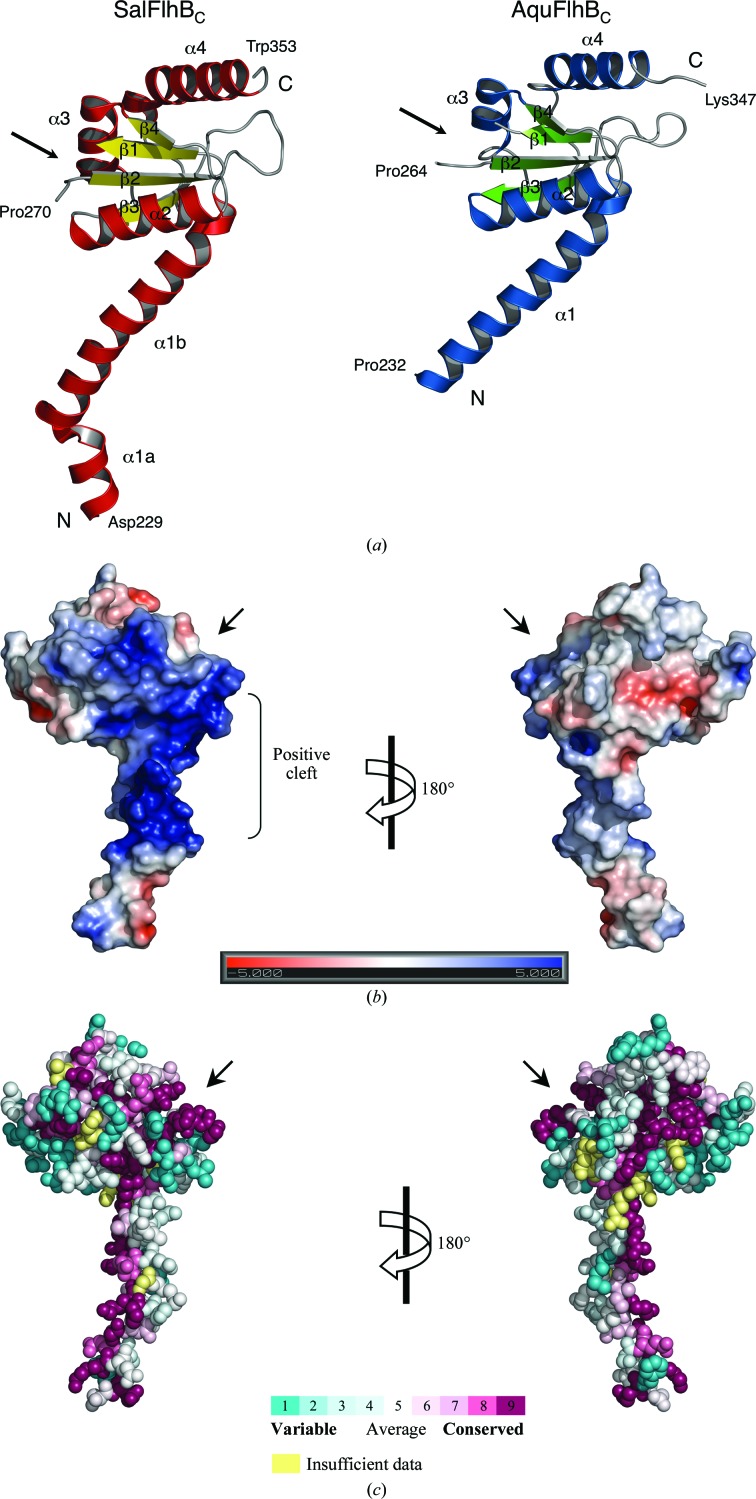
Structure of flagellar FlhB_c_. (*a*) Ribbon representation of the crystal structures of *Salmonella* and *Aquifex* FlhB_C_. (*b*) Electrostatic potential mapped onto the surface of *Salmonella* FlhB_C_. Electrostatics were calculated using the *APBS* software (Baker *et al.*, 2001 ▶) and plotted at ±5*kT* e^−1^. (*c*) Evolutionarily conserved residues of FlhB_C_. The figure was prepared with *ConSurf* (http://consurf.tau.ac.il/; Ashkenazy *et al.*, 2010 ▶). Residues are coloured according to the conservation in amino-acid sequences of 200 different FlhB proteins. Arrows mark the position of the autocleavage site between β1 and β2.

Both the *Salmonella* and *Aquifex* FlhB_C_ structures show very similar folds, with an r.m.s.d. of 1.03 Å for 102 C^α^ atoms (Fig. 2 ▶ *a*). Flagellar FlhB_C_ consists of a globular domain composed of a four-stranded β-sheet surrounded by four α-helices. The globular domain is preceded by a long N-terminal α-helix (α1) that connects the cytoplasmic globular part of FlhB to the transmembrane domain. The α1 helix engages in a crystal contact in both the *Salmonella* and *Aquifex* FlhB_C_ crystals, which may affect its orientation relative to the globular domain. However, these crystal contacts differ. In the SalFlhB_C_ crystal α1 primarily contacts α1 and α2 of adjacent molecules, while in the AquFlhB_C_ crystal α1 primarily contacts α4 and the cleavage site between β1 and β2.

The major difference between SalFlhB_C_ and AquFlhB_C_ is in the N-­terminal region. In the model of SalFlhB_C_ helix α1 is longer and has a kink at the highly conserved residue Gly236. However, a longer helix with a kink is not excluded in AquFlhB_C_, in which a highly conserved Gly230 occurs just two residues into the disordered segment 213–231 which is present in the crystallized protein but is absent in the model. Although the kink may arise from the crystal packing, our data show potential flexibility of the linker around this conserved glycine residue. The importance of such flexibility has previously been shown for EscU, an FlhB paralogue from the needle TTSS. Mutation of Gly229 (which corresponds to Gly236 of SalFlhB) to the less flexible proline in EscU completely abolished secretion (Zarivach *et al.*, 2008 ▶).

The conserved NPTH autocleavage site is exposed on a surface between strands β1 and β2. Both *Salmonella* and *Aquifex* FlhB_C_ show different conformations of the PTH region that suggest its flexibility. This is very different from the needle paralogues. In all known paralogue structures the PTH region has the same orientation, which is stabilized by contacts with surrounding residues (Zarivach *et al.*, 2008 ▶; Deane *et al.*, 2008 ▶; Wiesand *et al.*, 2009 ▶; Lountos *et al.*, 2009 ▶). It is difficult to say for the moment whether the greater flexibility of the PTH site in flagellar FlhB_C_ has any functional meaning. In SalFlhB_C_, the PTH region, together with adjacent residues in the globular domain and the C-terminal part of the linker α-helix, forms a positively charged cleft (Fig. 2 ▶ *b*). A similar positive cleft is also present in AquFlhB_C_. Such a cleft might be a potential recognition site for proteins secreted by the flagellar secretion system. The autocleavage of FlhB has been suggested to create an inter­action site for the other components of the type III secretion system (Zarivach *et al.*, 2008 ▶; Deane *et al.*, 2008 ▶). In particular, there is a model that describes the binding of FliK to the cleaved NPTH loop of FlhB (Mizuno *et al.*, 2011 ▶). However, the linker helix, which is one of the most conserved parts of the FlhB protein (Fig. 2 ▶ *c*), could also participate in the recognition of secreted proteins, since deletions or point mutations in this region of FlhB completely block secretion (Fraser *et al.*, 2003 ▶; Zarivach *et al.*, 2008 ▶).

### 3.2. Comparison with needle paralogue structures   

**Figure 3:**
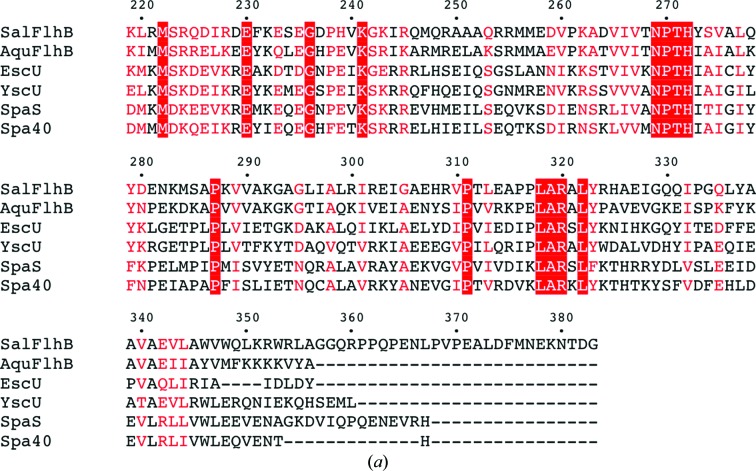

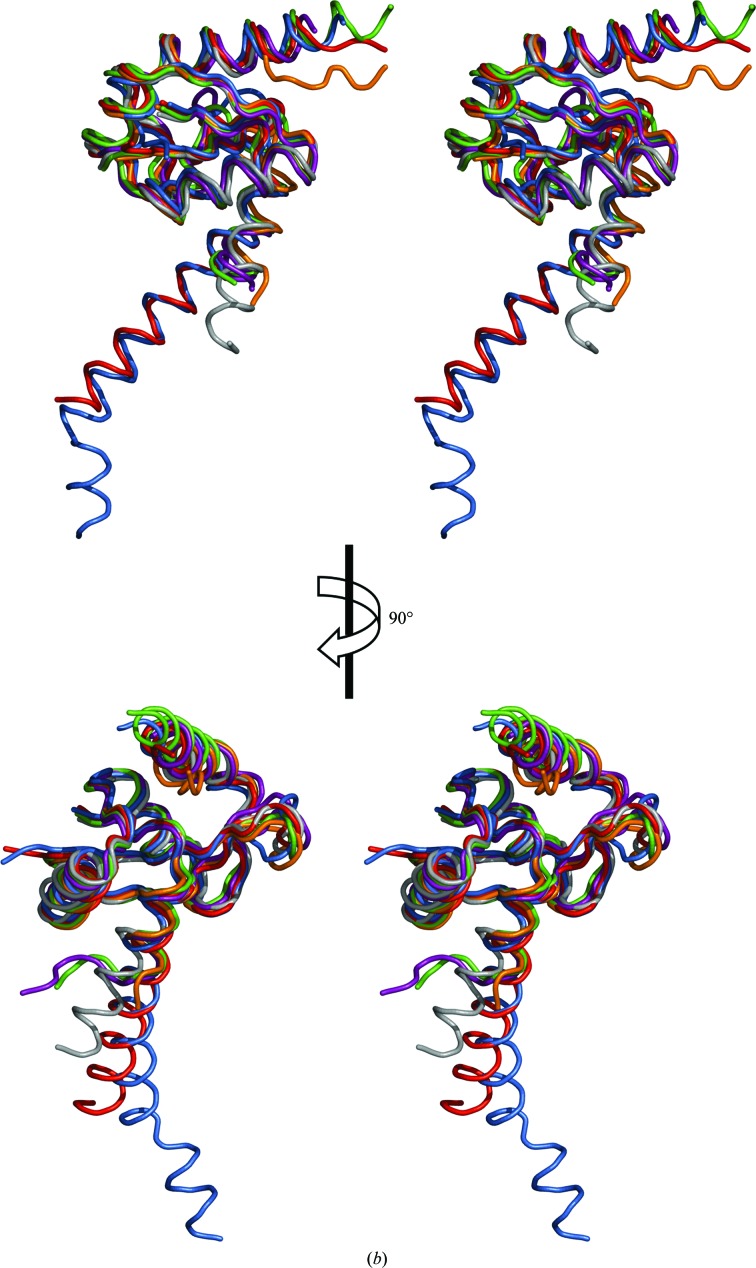
Comparison of flagellar FlhB_C_ and its paralogues from the needle type III secretion system. (*a*) Multiple sequence alignment of FlhB_C_ from *S. typhimurium* (SalFlhB_C_; Swiss-Prot accession No. P40727) with FlhB_C_ from *A. aeolicus* (AquFlhB_C_; O67813), EscU_C_ from *E. coli* (Q7DB59), YscU_C_ from *Yersinia pestis* (P69986), SpaS_C_ from *S. typhimurium* (P40702) and Spa40_C_ from *Shigella flexneri* (Q6XVW1). Identical residues are boxed in red; similar residues are coloured red. Alignment was performed with *ClustalW* (Larkin *et al.*, 2007 ▶; Goujon *et al.*, 2010 ▶). (*b*) Stereoviews of the superposition of *Salmonella* FlhB_C_ (blue; PDB entry 3b0z; this work), *Aquifex* FlhB_C_ (red; PDB entry 3b1s; this work), EscU_C_ (orange; PDB entry 3bzo; Zarivach *et al.*, 2008 ▶), YscU_C_ (grey; PDB entry 2jli; Lountos *et al.*, 2009 ▶), SpaS_C_ (green; PDB entry 3c01; Zarivach *et al.*, 2008 ▶) and Spa40_C_ (purple; PDB entry 2vt1; Deane *et al.*, 2008 ▶).

Despite low sequence identity (Fig. 3 ▶ *a*), the overall structure of flagellar FlhB_C_ is very similar to the structures of paralogues from the needle secretion system: EscU_C_, SpaS_C_, YscU_C_ and Spa40_C_ (Fig. 3 ▶ *b*). The obvious difference between these proteins is the linker region between the N-terminal transmembrane domain and the globular cytoplasmic domain. All of the proteins show a large difference in the conformation of their N-terminal parts, indicating flexibility of this region of the molecule. In our structures no electron density was observed for residues 219–228 of SalFlhB_C_ and residues 213–231 of AquFlhB_C_, which is consistent with flexibility of this part of FlhB_C_. However, the remainder of the residues of the linker form a well defined α-­helix, which in the case of SalFlhB_C_ is kinked at position Gly236. In contrast to the needle paralogues, it might be a general property of flagellar FlhB to have a more stable linker helix.

The proteins of the FlhB family exhibit a significant variation in length, mainly because of differences at the C-terminus. For instance, *Salmonella* FlhB is longer than the *Aquifex* protein by 33 amino acids. However, these additional residues (residues 354–383) were not visible in the electron-density map, suggesting that they are unfolded. This region in SalFlhB is rich in proline residues, making it unlikely to form any stable structure. The function of the elongated C-terminal part of FlhB is not known, but it is dispensable for motility (Kutsukake *et al.*, 1994 ▶). It apparently participates in the regulation of secretion because C-­terminal truncation of *Salmonella* FlhB can partially suppress the Δ*fliK* phenotype (Kutsukake *et al.*, 1994 ▶; Williams *et al.*, 1996 ▶). However, it is unlikely to directly interact with FliK since the truncation has almost no effect in a wild-type *fliK* background (Williams *et al.*, 1996 ▶).

### 3.3. Effect of mutations of residues 281–285 of *Salmonella* FlhB on TTSS function   

**Figure 4:**
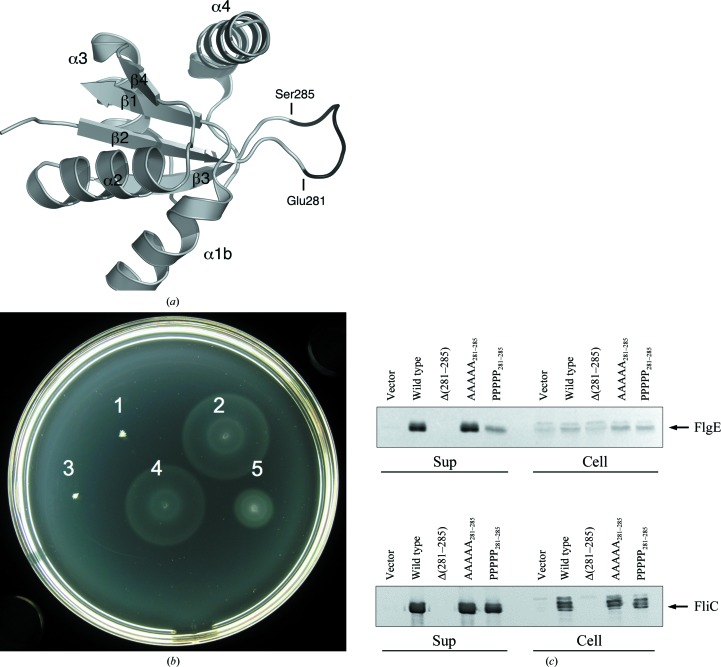
Effect of mutations of the ENKMS_281–285_ region of *Salmonella* FlhB on protein function. (*a*) Ribbon representation of *Salmonella* FlhB_C_; the region is shown in black. (*b*) The ability of FlhB variants with a modified ENKMS region to complement the Δ*flhB Salmonella* strain MKM50. Motility assays were carried out on semi-solid agar plates at 303 K for 5 h. FlhB products were 1, none, empty vector; 2, wild-type FlhB; 3, FlhB Δ(281–285); 4, FlhB AAAAA_281–285_; 5, FlhB PPPPP_281–285_. (*c*) Immunoblotting using anti-FlgE and anti-FliC antibodies on the whole-cell (Cell) and culture-supernatant (Sup) fractions from *Salmonella* strain MKM50 producing different FlhB variants.

The two strands β2 and β3 are connected by a long flexible loop. This loop is not conserved within the FlhB family, although it is flanked by highly conserved residues: Tyr279 and Pro287 (in *Salmonella* numbering). The length of the loop, which is longer than necessary just to connect two β-­strands, made us think that it might be of functional importance. To investigate this hypothesis, we created three mutants of *Salmonella* FlhB. In the first mutant the loop residues 281–285 were deleted (Fig. 4 ▶ *a*). In the second and third mutants residues 281–285 were substituted by Ala or Pro residues, respectively. We then carried out swarming assays on soft agar plates to investigate whether the *Salmonella* cells containing mutated FlhB were still motile. We found that the deletion of the loop completely abolished motility (Fig. 4 ▶ *b*). At the same time, substitution by Ala residues had no effect on motility and Pro substitution decreased motility. To check whether these changes in motility were because of changes in export activity, we analyzed secretion of the hook protein FlgE and the filament protein FliC by the flagellar secretion system containing mutated FlhB (Fig. 4 ▶ *c*). We found that motility is correlated to the secretion of FlgE and FliC. In the case in which the loop (281–285) of FlhB was deleted neither FlgE nor FliC were secreted, whereas proline substitution reduced the secretion of both proteins. No difference in secretion was observed between the wild-type FlhB and the Ala substitution.

### 3.4. Molecular-dynamics (MD) simulations   

**Figure 5:**
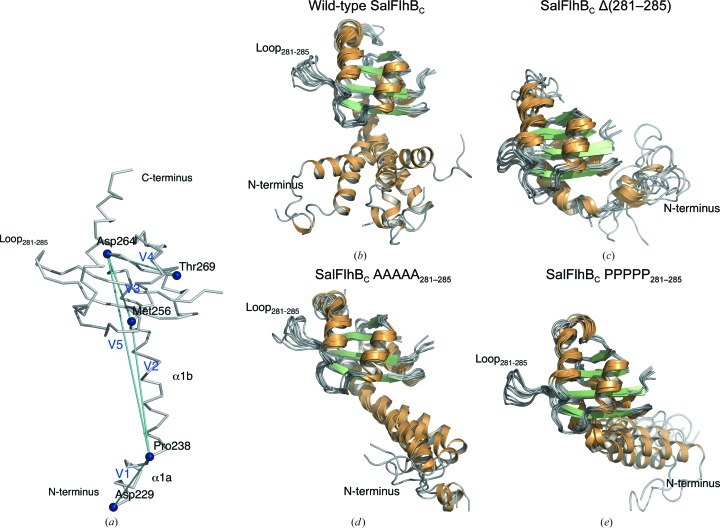
Flexibility of the N-terminal α-helix of *Salmonella* FlhB_C_ observed in MD simulations. (*a*) Key residues and vectors used for MD analysis in Table 2 ▶. The vectors connecting residues 229–238, 238–256, 256–264, 265–269 and 238–264 are defined as V1–V5, respectively. (*b*–*e*) Structural variations of the N-­terminal α-helix during MD simulations when the globular domain is superimposed for (*b*) the wild-type FlhB_C_, (*c*) FlhB_C_ Δ(281–285), (*d*) FlhB_C_ AAAAA_281–285_ and (*e*) FlhB_C_ PPPPP_281–285_. For each case, six snapshots were chosen based on the maximum and minimum values of *D*, θ_14_ and χ_5_ (see Table 2 ▶ for their definitions).

To further investigate the effect of mutation of the loop on the FlhB_C_ molecule, we performed MD simulations of the wild-type SalFlhB_C_ and the Δ(281–285), AAAAA_281–285_ and PPPPP_281–­285_ mutants. During the MD simulations, we observed that the globular domain is relatively rigid in all of the cases, while the N-­terminal α-helix of the wild-type FlhB_C_ is very flexible and becomes less flexible in the mutants (Figs. 5 ▶ *b*–5*e*). In addition to the kink around Gly236–Pro238 (Fig. 2 ▶ *a*), a significant kink was observed near Met256 during the MD simulations. To characterize the flexibility of the N-­terminal α-helix, we defined a distance *D*, angles θ_12_, θ_23_, θ_34_ and θ_14_ and torsion angles χ_3_ and χ_5_ (see explanations in Fig. 5 ▶ *a* and Table 2 ▶). A notable structural difference is demonstrated by torsion angle χ_3_, which determines the direction of the V2 region of the N-­terminal α-helix relative to the globular domain. The χ_3_ value is positive for wild-type FlhB_C_ and the AAAAA_281–285_ mutant, but is negative for the Δ(281–285) and PPPPP_281–285_ mutants, which is consistent with the structural differences shown in Figs. 5 ▶(*b*)–5(*e*). Since the latter mutations reduce *Salmonella* motility, this structural change might have some functional effects. Another notable difference is observed as a reduction in the θ_12_, θ_23_, θ_14_, χ_3_ and χ_5_ fluctuations in the PPPPP_281–285_ mutant, indicating significant structural change and loss of flexibility of the N-terminal α-­helix.

## 4. Conclusions   

The flagellar secretion system is closely related to the needle type III secretion system utilized by a number of virulent bacteria for the secretion of toxins into the host-cell cytoplasm. The structure of flagellar FlhB_C_ described in this paper once again confirms this relationship. Basically, the structure is similar to already known structures of needle paralogues. The differences (such as the orientation of the conserved PTH sequence or the longer linker helix) could be peculiarities of the flagellar protein. The functional implications of such differences, if any, are a subject for further investigation.

The most important of our findings is that flexibility of the large nonconserved loop in the globular domain of FlhB_C_ is necessary for function of the whole secretion system. Deletion of this loop or its mutation to less flexible proline residues makes FlhB_C_ more rigid and thus abolishes or significantly reduces secretion. Taking into account the similarity between the flagellar and needle proteins, this loop could be a promising target for the creation of novel drugs against pathogenic bacteria.

## Supplementary Material

PDB reference: *Salmonella* FlhB_C_, 3b0z

PDB reference: *Aquifex* FlhB_C_, 3b1s

## Figures and Tables

**Table 1:** X-ray data-collection and refinement statistics Values in parentheses are for the highest resolution shell. MAD data-collection statistics for *Salmonella* FlhB_C_ have been published in Meshcheryakov Samatey (2011 ▶).

	*Salmonella* FlhB_c_	*Aquifex* FlhB_c_			
	Native	Native	SeMet derivative		
			Peak	Inflection	Remote
Data collection					
Space group	*P*4_2_2_1_2	*C*2	*C*2		
Unit-cell parameters (, )	*a* = *b* = 49.1, *c* = 143.1, = = = 90	*a* = 114.6, *b* = 33.8, *c* = 122.4, = = 90, = 107.8	*a* = 113.4, *b* = 33.6, *c* = 122.2, = = 90, = 107.9		
Molecules in asymmetric unit	1	3	3		
Wavelength ()	0.9	0.9	0.9791	0.97936	0.99508
Resolution ()	40.452.45 (2.582.45)	47.762.55 (2.692.55)	503.00 (3.163.00)	503.00 (3.163.00)	503.00 (3.163.00)
*R*_merge_ †	0.075 (0.380)	0.056 (0.386)	0.094 (0.452)	0.069 (0.407)	0.064 (0.368)
*I*/(*I*)	16.2 (5.7)	12.5 (3.4)	7.6 (2.4)	9.9 (3.0)	10.5 (3.2)
Completeness (%)	98.8 (100)	99.3 (100)	100 (100)	100 (100)	100 (100)
Multiplicity	7.7 (7.9)	3.7 (3.8)	3.6 (3.7)	3.7 (3.7)	3.7 (3.7)
Refinement					
Resolution ()	28.072.45	29.752.55			
*R*_work_/*R*_free_ (%)	23.1/24.7	24.1/26.2			
No. of atoms					
Protein	992	2707			
Ligand/ion	4	0			
Water	20	48			
Wilson plot *B* factor (^2^)	79.3	83.7			
Average *B* factor (^2^)					
Protein	78.8	73.6			
Ligand/ion	77.2	N/A			
Water	39.3	61.2			
R.m.s. deviations					
Bond lengths ()	0.021	0.019			
Bond angles ()	2.090	1.844			
Ramachandran plot (%)					
Most favoured	97.5	99.7			
Additionally allowed	2.5	0.3			
Disallowed	0	0			

† *R*_merge_ = 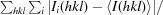 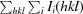, where *I_i_*(*hkl*) is the intensity of the *i*th measurement of reflection *hkl* and *I*(*hkl*) is the mean value of *I_i_*(*hkl*) for all *i* measurements.

**Table 2:** Structure and fluctuation differences between wild-type *Salmonella* FlhB_C_ and its mutants during MD simulation shown by the key distance and angles defined by vectors V15 shown in Fig. 4 ▶(*a*) *D*, length of V5. _12_, _23_, _34_ and _14_, angles defined between V1 and V2, V2 and V3, V3 and V4, and V1 and V4, respectively. _3_ and _5_, torsion angles defined by the sets of three vectors V2V3V4 (torsion around V3) and V1V5V4 (around V5), respectively. Averages and standard deviations over the last 20ns of MD are shown, with negative values shown in bold.

Protein	*D* ()	_12_ ()	_23_ ()	_34_ ()	_14_ ()	_3_ ()	_5_ ()
*Salmonella* FlhB_C_							
Wild type	37.2 3.9	96.0 21.9	53.8 19.4	105.5 2.2	77.1 38.3	83.8 23.5	**103.8 44.8**
(281285)	36.9 0.7	133.2 11.7	64.1 5.2	108.2 1.7	34.0 9.5	**23.1 7.9**	168.3 21.0
AAAAA_281285_	38.1 1.5	113.3 34.6	45.7 6.1	113.2 1.8	47.9 35.4	30.3 11.6	**175.5 43.6**
PPPPP_281285_	36.6 3.1	69.6 37.1	51.4 12.2	112.9 2.4	59.2 26.9	**77.3 18.9**	**165.4 43.1**
